# Genetic markers associated with the widespread insecticide resistance in malaria vector *Anopheles funestus* populations across Tanzania

**DOI:** 10.1186/s13071-024-06315-4

**Published:** 2024-05-17

**Authors:** Joel O. Odero, Ismail H. Nambunga, John P. Masalu, Gustav Mkandawile, Hamis Bwanary, Emmanuel E. Hape, Rukiyah M. Njalambaha, Patrick Tungu, Halfan S. Ngowo, Emmanuel W. Kaindoa, Salum A. Mapua, Najat F. Kahamba, Luca Nelli, Charles Wondji, Lizette L. Koekemoer, David Weetman, Heather M. Ferguson, Francesco Baldini, Fredros O. Okumu

**Affiliations:** 1 Environmental Health and Ecological Sciences Department, Ifakara Health Institute, P.O. Box 53, Ifakara, Tanzania; 2 School of Biodiversity, One Health, and Veterinary Medicine, University of Glasgow, Glasgow, G12 8QQ UK; 3 School of Life Science and Biotechnology, Nelson Mandela African Institution of Science and Technology, P.O. Box 447, Arusha, Tanzania; 4 Amani Medical Research Centre, National Institute for Medical Research, Muheza, Tanzania; 5 Wits Research Institute for Malaria, Faculty of Health Sciences, University of the Witwatersrand, 7 York Road, Parktown, Johannesburg, South Africa; 6 Centre for Emerging Zoonotic & Parasitic Diseases, National Institute for Communicable Diseases, A Division of the National Health Laboratory Service, Johannesburg, South Africa; 7 Department of Vector Biology, Liverpool School of Tropical Medicine, Liverpool, L3 5QA UK; 8 Department of Medical Entomology, Centre for Research in Infectious Diseases (CRID), Yaoundé 5, Cameroon

**Keywords:** Insecticide resistance, *Anopheles funestus*, *CYP6P9a/b*, *L119F-GSTe2*, Structural variants, Vector surveillance, Tanzania

## Abstract

**Background:**

*Anopheles funestus* is a leading vector of malaria in most parts of East and Southern Africa, yet its ecology and responses to vector control remain poorly understood compared with other vectors such as *Anopheles gambiae* and *Anopheles arabiensis*. This study presents the first large-scale survey of the genetic and phenotypic expression of insecticide resistance in *An. funestus* populations in Tanzania.

**Methods:**

We performed insecticide susceptibility bioassays on *An. funestus* mosquitoes in nine regions with moderate-to-high malaria prevalence in Tanzania, followed by genotyping for resistance-associated mutations (*CYP6P9a, CYP6P9b, L119F-GSTe2*) and structural variants (SV4.3 kb, SV6.5 kb). Generalized linear models were used to assess relationships between genetic markers and phenotypic resistance. An interactive R Shiny tool was created to visualize the data and support evidence-based interventions.

**Results:**

Pyrethroid resistance was universal but reversible by piperonyl-butoxide (PBO). However, carbamate resistance was observed in only five of the nine districts, and dichloro-diphenyl-trichloroethane (DDT) resistance was found only in the Kilombero valley, south-eastern Tanzania. Conversely, there was universal susceptibility to the organophosphate pirimiphos-methyl in all sites. Genetic markers of resistance had distinct geographical patterns, with *CYP6P9a*-R and *CYP6P9b*-R alleles, and the SV6.5 kb structural variant absent or undetectable in the north-west but prevalent in all other sites, while SV4.3 kb was prevalent in the north-western and western regions but absent elsewhere. Emergent *L119F-GSTe2*, associated with deltamethrin resistance, was detected in heterozygous form in districts bordering Mozambique, Malawi and the Democratic Republic of Congo. The resistance landscape was most complex in western Tanzania, in Tanganyika district, where all five genetic markers were detected. There was a notable south-to-north spread of resistance genes, especially *CYP6P9a*-R, though this appears to be interrupted, possibly by the Rift Valley.

**Conclusions:**

This study underscores the need to expand resistance monitoring to include *An. funestus* alongside other vector species, and to screen for both the genetic and phenotypic signatures of resistance. The findings can be visualized online via an interactive user interface and could inform data-driven decision-making for resistance management and vector control. Since this was the first large-scale survey of resistance in Tanzania’s *An. funestus*, we recommend regular updates with greater geographical and temporal coverage.

**Graphical Abstract:**

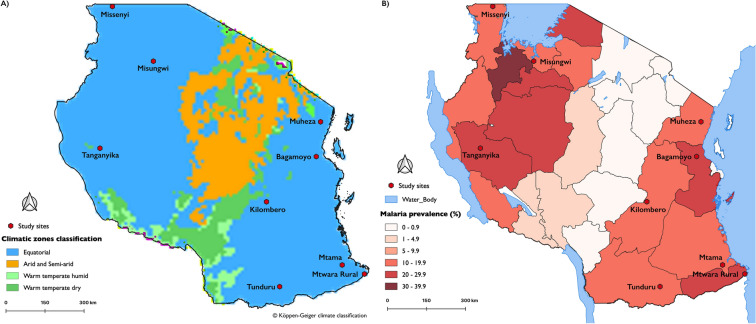

## Background

Vector control constitutes one of the key pillars of malaria control in Africa [1], where it primarily constitutes the deployment of insecticide-treated nets (ITNs) and indoor residual spraying (IRS). Despite great successes, in particular since 2000, malaria remains a significant challenge [2], partly due to biological threats, notably the evolution and spread of insecticide resistance [3, 4]. To accelerate progress, affected countries must adopt proactive strategies to manage widespread resistance and optimize the allocation of insecticidal interventions.

In the east and southern African region, malaria transmission is mediated primarily by *Anopheles funestus* [5], which, like *Anopheles gambiae*, is highly adapted to human habitations [6]. In Tanzania, studies in the high prevalence settings in the north-east and south-eastern regions have shown that this vector species can mediate as high as 90% of the local malaria infections or more [7, 8]. Unfortunately, compared with *An. gambiae* and *Anopheles arabiensis,* for which there have been several insecticide resistance surveys in Tanzania [9], only a handful of studies have profiled the resistance patterns in *An. funestus* [7, 8, 10, 11]. This lack of data, especially the phenotypic data, can be attributed to difficulties associated with rearing *An. funestus* and identifying its aquatic habitats [12, 13], which complicates the collection of age-synchronized larvae required for age-standardized resistance bioassays [14]. The resulting information gap can derail the effective planning, implementation and monitoring of vector control programs in settings where *An. funestus* dominates.

Comprehensive information on molecular mechanisms driving resistance in *An. funestus* in Tanzania is similarly scarce [11]. Unlike phenotypic bioassays, the genetic signals of insecticide resistance can enable early detection and mitigation before the problem is widely established in vector populations [15]. By establishing the exact mechanisms underlying the observed phenotypes, molecular monitoring also enables the elucidation of causes and risk factors of resistance in specific locations. However, establishing direct correlations between the phenotypes and molecular resistance can be challenging due to the vast number of mechanisms and gene families, as well as the stochastic nature of the genetic interactions leading to resistance [16, 17]. In *An. funestus*, the cytochrome P450 (*CYP450*) monooxygenases gene family constitutes the dominant family of pyrethroid resistance genes and has been associated with reduced efficacy of pyrethroid-based ITNs [18]. Notably, *CYP6P9a* and *CYP6P9b* are highly expressed in pyrethroid-resistant *An. funestus* populations in east and southern Africa [19], leading to operational failures in vector control in the region [20]. Another cytochrome P450 gene, *CYP9K1*, is significantly overexpressed in pyrethroid-resistant *An. funestus* populations from Uganda [21, 22], while *CYP6P4a* and *CYP6P4b* have been found in Ghana [23]. While many of these genes are associated with resistance to a narrow range of insecticides, certain mutations can confer resistance to multiple insecticide classes. For instance, the L119F mutation in Glutathione S-Transferases epsilon 2 (*GSTe2*) facilitates broad detoxification of pyrethroids, organochlorides and organophosphates in *An. funestus* [24]. Until now, genetic analysis of *L119F-Gste2* in field-collected mosquitoes is geographically restricted to west and central Africa [24–27], with its spread resistance role in east and southern Africa remaining unclarified.

Novel IR mechanisms such as copy number variants have also recently been demonstrated to increase the expression of insecticide-detoxifying genes, leading to resistance in *An. funestus* [28, 29]. Monitoring the evolution and spread of these resistance genes in the face of sustained pressure from insecticide-based vector control is vital to managing resistance. Fortunately, field-adaptable molecular assays are now available that can track some molecular resistance genes in *An. funestus* [18, 19, 26, 28, 29], and can be leveraged to monitor resistance and inform appropriate deployment of the limited vector control tools.

In this study, we comprehensively analysed the insecticide resistance phenotypes and genotypes *An. funestus* mosquitoes in nine regions representing different eco-epidemiological settings in Tanzania. We aimed to understand the potential responsiveness of these mosquitoes to public health insecticides, generate insights for more effective strategies for malaria control, and establish a baseline chart of insecticide resistance in Tanzania’s *An. funestus*. We screened for five major genes and structural variants known to confer metabolic resistance and conducted standard susceptibility tests against pyrethroids, carbamates, organophosphates and organochlorides. This study represents the first large-scale survey of the insecticide resistance patterns in *An. funestus*, which despite being a dominant vector in the country, remains far less investigated compared with other malaria vectors.

## Methods

### Study area and mosquito collection

**Fig. 1:**
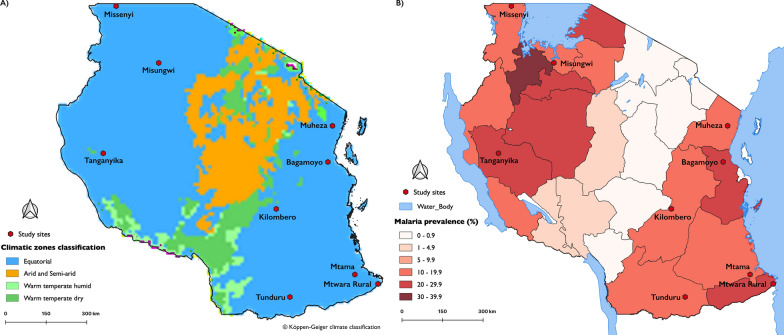
Map of Tanzania showing districts (red dots) selected for insecticide resistance surveillance. **A** Sites were selected on the basis of sentinel data from Tanzania’s national malaria control program that confirmed the presence of *An. funestus* group mosquitoes (all sites) and **B** national malaria prevalence data encompassing moderate (10–19.9%) (Missenyi, Misungwi, Kilombero, Tunduru and Muheza) and high (> 20%) (Tanganyika, Mtwara rural, Bagamoyo and Mtama) malaria burden. Malaria prevalence data were adopted from the Tanzanian Ministry of Health report on the School Malaria and Nutrition Survey (SMNS) [36]

Mosquitoes were collected from November 2021 to December 2022 in selected villages across nine administrative districts of Tanzania (Fig. 1). The locations represent a comprehensive geographical and epidemiological cross-section of the country and were selected for their high prevalence of *An. funestus* [30]. Due to difficulties in finding *An. funestus* immature stages and unsuccessful attempts to get sufficient offspring from wild blood-fed females, we adopted a previously tested approach relying on unfed and non-gravid females of unknown ages for the resistance bioassays [8, 10]. Following consent of household heads, adult mosquitoes were sampled inside houses using a combination of centres for disease control and prevention (CDC)-miniature light traps (CDC-LT) [31] and a miniaturized double-net trap (DN-Mini) [32]. The collected mosquitoes were first sorted by taxa using taxonomic keys [33–35], and the *An. funestus* mosquitoes were further sorted on the basis of physiological features [33–35] and abdominal status (unfed). The unfed and non-gravid females were maintained ad libitum on 10% sucrose-soaked cotton wool for a day to acclimatize and select weak individuals not suitable for the resistance tests.

### Tests for susceptibility of Anopheles funestus to different insecticides

The acclimatized mosquitoes were sugar-starved for at least 2 hours before being utilized in the bioassays, following the guidelines for World Health Organization (WHO) tube tests for mosquito susceptibility [14]. Room temperature and relative humidity were recorded at the time of each test. The candidate insecticides included those commonly used for vector control either on ITNs (permethrin, piperonyl butoxide and deltamethrin) or for IRS (bendiocarb, pirimiphos-methyl,) [30]. In addition, we included dichloro-diphenyl-trichloroethane (DDT), the persistent organic pollutant, which is no longer used for vector control in Tanzania [37].

The susceptibility of adult *An. funestus* mosquitoes were determined by exposing the acclimatized females for 60 min to WHO-approved insecticide-impregnated papers containing the diagnostic doses of the candidate insecticides: bendiocarb (0.1%), pirimiphos-methyl (0.25%), DDT (4%), permethrin (0.75%) and deltamethrin (0.05%). For each round of the bioassays and each insecticide, we included four test tubes lined with the impregnated papers and two control tubes lined with untreated control papers containing respective solvent oils. A total of 20 mosquitoes were exposed in each tube. The tests were thus conducted in four test replicates with two control tubes. After the 1-hour exposure and knock-down monitoring, the mosquitoes were maintained in insecticide-free tubes for 24 h to monitor mortality.

### Tests for the intensity of pyrethroid resistance in Anopheles funestus

Intensity assays are generally used to determine the operational significance of the resistance phenotypes once resistance is confirmed using the discriminating insecticide doses. In line with WHO guidelines, the intensity of resistance against the two pyrethroids was determined by exposing the mosquitoes to increasing concentrations of the insecticides. We used either 5× and 10× the doses of permethrin (i.e. 3.75% and 7.5%, respectively) and 5× and 10× the doses of deltamethrin (i.e. 0.25% and 0.5%, respectively). Knockdown was recorded after 1 h following insecticide exposure and mortality was recorded after a further 24 h of monitoring. The tests were also conducted in four test replicates with two control tubes in which mosquitoes were exposed to papers containing silicone oil but no insecticides.

### Tests of the synergistic effect of piperonyl butoxide (PBO) on pyrethroid-resistant Anopheles funestus

Piperonyl butoxide (PBO) is a synergist commonly used on ITNs to enhance their insecticidal activity on resistant mosquitoes by inhibiting the function of cytochrome P450 monooxygenases. In this study, the role of PBO in reversing pyrethroid resistance phenotype was determined by first exposing the female *An. funestus* mosquitoes to 4% PBO for 1 h, followed by a 1-h exposure to the diagnostic dose of deltamethrin (0.05%). The tests were conducted in three replicate tubes with two control tubes, in which mosquitoes were exposed to control papers containing only the solvent oil. Knockdown was recorded after 1 h following insecticide exposure and mortality was recorded after a further 24 h of monitoring [14].

### Tests to confirm the genetic identity of the Anopheles funestus mosquitoes.

To enhance the probability of the collected mosquitoes being *An. funestus* s.s., rather than the other species within the *An. funestus* group, which comprises at least 11 known members [34, 35], the field collections had been strategically conducted in villages with high densities of *An. funestus s.s*. The collections were specifically done indoors, aligning with the species’ endophilic behaviour. Nonetheless, to confirm their genetic identity, polymerase chain reaction (PCR) assays were performed on the mosquitoes once the resistance assays had been completed.

A subset of the mosquitoes (~ 20%) that were either alive or dead after 24 h following insecticide exposure were randomly selected from the replicates and preserved in 80% ethanol. Genomic DNA was extracted from individual mosquitoes using a DNeasy Blood & Tissue Kit (Qiagen, UK). Species identification followed a PCR-based protocol utilizing species-specific primers targeting the non-coding internally transcribed spacer region (ITS2) between the 5.8S and 28S ribosomal DNA sequence to distinguish between at least seven members of *An. funestus* group [38, 39]. Briefly, 0.33 µl of each 10 µm primer was added to 6.25 µl of 2X GoTaq® G2 Green Master Mix (Promega, USA) in a 20 µl reaction volume. The cycling conditions were as follows: 5 min at 95 °C followed by 30 cycles of 94 °C denaturation, 45 °C annealing and 72 °C extension, and a final extension at 72 °C for 5 min. The PCR products were separated on a 2% agarose gel stained with SYBR™ Safe DNA Gel Stain (Invitrogen, UK).

In addition to testing the subset of mosquitoes used for the actual resistance bioassays, we also tested a larger sub-sample of mosquitoes originating from a broader field survey that had been completed in these same areas to provide more representative data on the diversity of the *An. funestus* group.

### Genotyping of selected metabolic resistance genes and structural variants (SV)

A total of 20 randomly selected mosquitoes that were either alive [10] or dead [10] 24 h post-exposure to discriminating insecticide doses of deltamethrin were identified by PCR to species level and genotyped to screen for the insecticide resistance genes *L119F-GSTe2*, *CYP6P9a* and *CYP6P9b*, as well as structural variants SV4.3 kb and SV6.5 kb, known to be associated with the overexpression of the resistance genes [28, 29]. Increased expression of these genes, or transposons in the case of SV, has been associated with resistance phenotypes in *An. funestus* [18, 19, 29].

The *L119F-GSTe2* mutation was genotyped following an allele-specific PCR protocol using two inner and outer primer pairs resulting in two fragments (850 bp and 312 bp) for the susceptible mosquitoes and three bands (850 bp, 12 bp and 523 bp) for the resistant haplotype [26]. The *CYP6P9a* gene was genotyped following a PCR assay targeting a restriction fragment length polymorphism (RFLP), where a restriction enzyme *Taq*I-V2 cuts a 450 bp fragment into 350 and 100 bp in mosquitoes carrying the resistance alleles but not in susceptible where the haplotypes remain uncut [18]. A similar assay was used for detecting *CYP6P9b*, but with restriction enzyme Tsp45I, which digests a 550 bp fragment into 400 bp and 150 bp in susceptible mosquitoes whereas resistant haplotypes remain uncut [18]. Finally, the detection of SV 6.5 kb and 4.3 kb was performed following methods described by Mugenzi et al. [28, 29]. Mosquitoes carrying the SV 6.5 kb variant had an amplicon fragment size of 569 bp for positive samples, 266 bp for negative samples, and both fragment sizes in heterozygotes. Similarly, the presence of the SV 4.3 kb variant was indicated by a 780 bp amplicon in positive individuals and a 280 bp amplicon in negatives, and both amplicons were observed in heterozygous individuals.

### Data analysis

In the initial susceptibility tests, we assessed the observed mortality in both treatment and control tests and used Abbott’s mortality adjustments in cases where control mortality exceeded ≥ 5% [14]. Tests were discarded if control mortality was 20% or more. The data were summarised, and mosquito populations were considered susceptible to an insecticide if mean mortality was ≥ 98% and resistant if mean mortality was ≤ 90% mortality [14]. In the resistance intensity assays, resistance was recorded as low if 24-h mortality at 5× was between ≥ 98%, moderate if mortality was below ≤ 98% at 5× but above ≥ 98% at 10× the dose, and high if 10× mortality was below 98%.

Generalised linear mixed models within the *lme4* package [35] were used to analyse the phenotypic resistance levels against different insecticides in R statistical software (version 4.1.1). Since these tests were done in different study sites, it was not possible to maintain a standard microclimatic condition in all cases, and as such, statistical analysis was used to discern the potential influence of these factors on the mortality outcomes. Mortality to each candidate insecticide was determined at the group level (i.e. averages of replicates for a given insecticide) in a binomial model with the response as the number of dead out of the number exposed and explanatory variables insecticide type (including controls), location of the test (district), temperature and relative humidity (RH) recorded at the time of test and interaction between insecticide and district; replicates were fitted as a random effect. The binomial model for the association between phenotype and the IR genotypes had mortality (alive/dead) response variable and explanatory variables as either resistant or susceptible genotypes of the five genes (*CYP6P9a, CYP6P9b, L119F-GSTe2,* SV 6.5 kb and SV 4.3 kb). *Drop1* command was used to examine the models and determine the variable to remove in a stepwise format starting with the most complex model. Additionally, the likelihood ratio test (LRT) was used to compare two nested models. The R command *ggemmeans* was used to run the model, and *gglot2* was used to plot the predictions. The variation in frequency of the five resistance genes by district was determined by analysis of variance (ANOVA) test on two logistic regressions, one with genes as the response and district as the predictor, and the other with just the intercept term.

## Results

### Confirmation of the molecular identity of the mosquitoes tested

Overall, PCR analysis showed that the proportions of *An. funestus* s.s in the sub-sample of mosquitoes collected in the nine districts (*N* = 6724 *An. funestus* group females) were as follows: Bagamoyo 89.8%, Kilombero 98.7%, Missenyi 99.4%, Misungwi 91.3%, Mtama 95.5%, Mtwara Rural 89.7%, Muheza 91%, Tanganyika 80.5% and Tunduru 97.7%. The residual proportions from these represented other sibling species within the *An. funestus* group, which included *An. leesoni, An. rivulorum, An. parensis* and *An. funestus/An leesoni* hybrid.

However, the mosquitoes used in the insecticide resistance bioassays were identified by PCR to be 100% *An. funestus *s.s. in all the study locations except in Tanganyika District, Katavi region in western Tanzania, where 15% were *An. parensis*. In Misungwi district, Mwanza region in north-western Tanzania, more than 90% of the mosquitoes of the samples were found to be *An. parensis* in the initially selected village (Ngaya), leading to the exclusion of this dataset and relocation to an alternative village with a higher occurrence of *An. funestus* (Nyang’omango). These notable instances of unusually high prevalences of *An. parensis* are discussed separately in an upcoming publication by Mapua et al. (unpublished).

All subsequent genetic screening for insecticide resistance markers for all study sites was performed only on *An. funestus *s.s. from the resistance tests and did not include any other sibling species in the *An. funestus* group.

### Resistance of An. funestus to pyrethroids

**Fig. 2:**
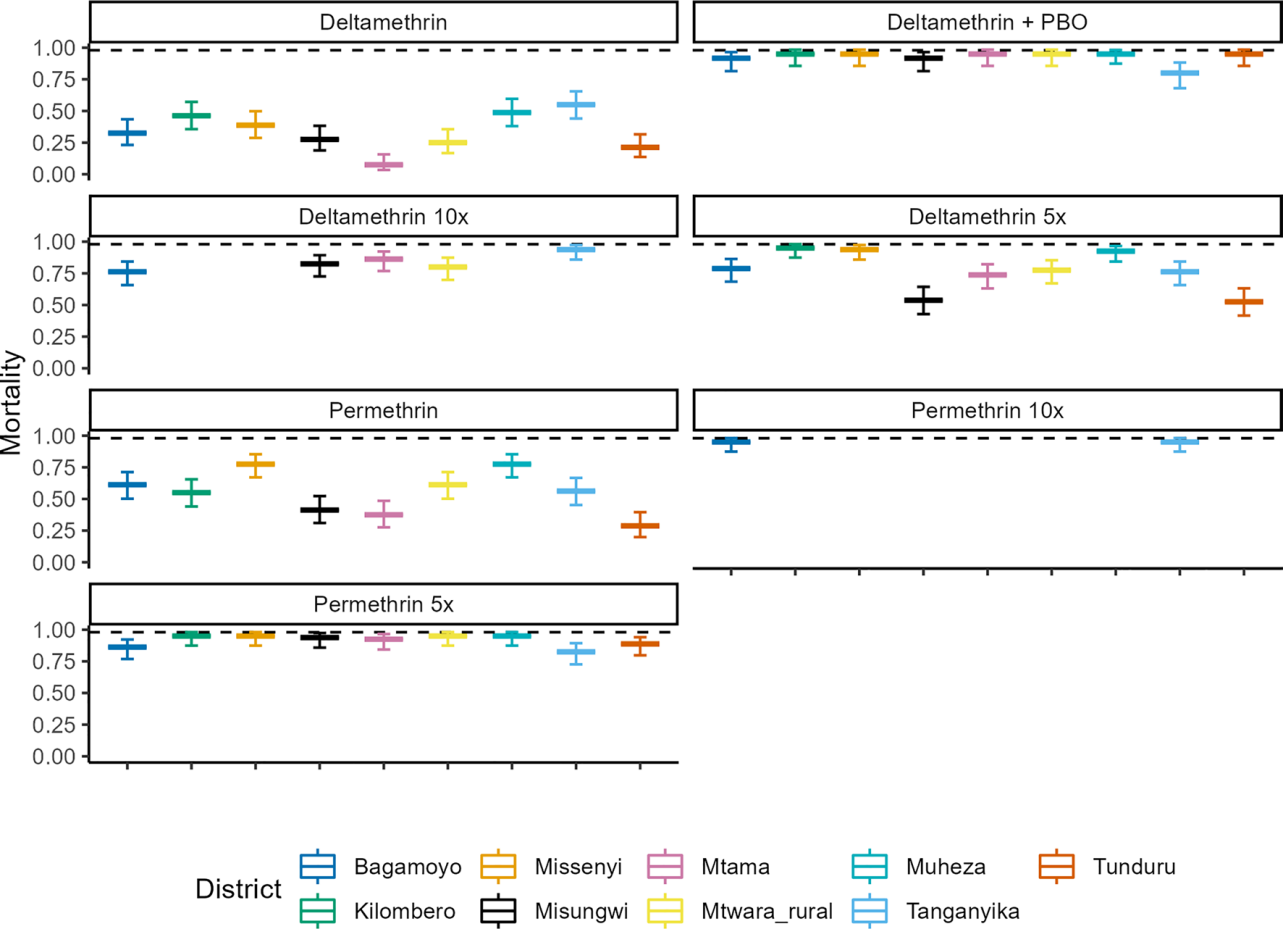
Insecticide resistance profile of *An. funestus:* Figure shows resistance to the discriminating insecticide doses, the intensity of resistance as observed in tests at 5× and 10× the diagnostic doses, and the synergistic effect of PBO in reversing the observed resistance. The colours represent the different districts where the bioassays were conducted, the error bars represent the 95% confidence intervals of the predictions and the dotted line on the *y*-axis indicates a 98% mortality threshold

*An. funestus* mosquitoes showed resistance to pyrethroid insecticides in all study sites, and 1-h knock-down (KD_1h_) to permethrin was lowest in Morogoro (35%) and highest in Muheza district (84%), while KD_1h_ deltamethrin was lowest in Mtama district 9% and highest in Tanganyika (48%). In tests against permethrin, the lowest 24-h mortality was 29% (confidence interval, CI 19.8–39.6) in Tunduru district, Ruvuma region in southern Tanzania, while the highest mortality was 76% (CI 67.1–85.4) in Missenyi district, in Kagera region, north-western Tanzania. Furthermore, in tests against deltamethrin, the lowest mortality was 7% (3.4–15.7) in Mtama district, Lindi region in south-eastern Tanzania, while the highest was 55% (CI 43.9–65.5) in Tanganyika district, Katavi region in western Tanzania (Fig. 2). In the analysis of factors influencing the observed mortality, we observed that the percentage mortality observed against the candidate insecticides varied between sites (insecticide × site interaction: *χ*^2^ = 187.4, *P* < 0.00005) but was not influenced by temperature (*χ*^2^ = 0.6168, *P* = 0.4345) or relative humidity (*χ*^2^ = 0.2326, *P* = 0.6296).

### Intensity of resistance to pyrethroids

The intensity of pyrethroid resistance was determined as low if 24-h mortality at the 5× dose was between ≥ 98%, moderate if the mortality was below 98% at 5× but above ≥ 98% at 10× the dose, and high if 10× mortality is < 98%. KD_1h_ was greater than 80% in all locations for 5× and 10× permethrin, lowest in Misungwi district (34%) and highest in Tanga (91%) for 5× deltamethrin and ranged 35% in Mtwara Rural and 84% in Tanganyika for 10× deltamethrin. In tests using permethrin, high-intensity resistance was observed in the coastal region, in Bagamoyo district [5× mortality: 86% (76.1–92.4); 10× mortality: 95% (87.4–98.1)], and Tanganyika district in the western part of the country [5 × mortality: 83% CI (72.5–89.3); 10× mortality: 95% (87.4–98.1)]. The rest of the study sites had only low-intensity resistance to this insecticide. In contrast, the resistance to deltamethrin was of high intensity in five out of the nine study sites districts. These included Bagamoyo, Mtama and Mtwara rural districts in eastern Tanzania; Tanganyika district in western Tanzania; and Misungwi district in the north of the country, where 24-h mortality was below 98% even at 10× the deltamethrin dose (Fig. 2).

### Synergistic effect of PBO and the reversal of pyrethroid resistance

Pre-exposure of the mosquitoes to PBO (synergist) for 1 h, followed by exposure to the candidate insecticide, resulted in the reversal of the initially observed resistance to the discriminating doses of deltamethrin (Fig. 2), and 1-h knock-down (KD_1h_) was greater than 98% in all locations. The resulting 24-h mortality in the PBO pre-exposed mosquitoes was greater than 98% in all sites except in Tanganyika, where it was only 80% CI (68–88.3). Due to limited supplies and the difficulty of obtaining sufficient mosquitoes, these PBO tests were conducted only for deltamethrin.

### Resistance of *An. funestus* to bendiocarb, DDT and pirimiphos-methyl

**Fig. 3:**
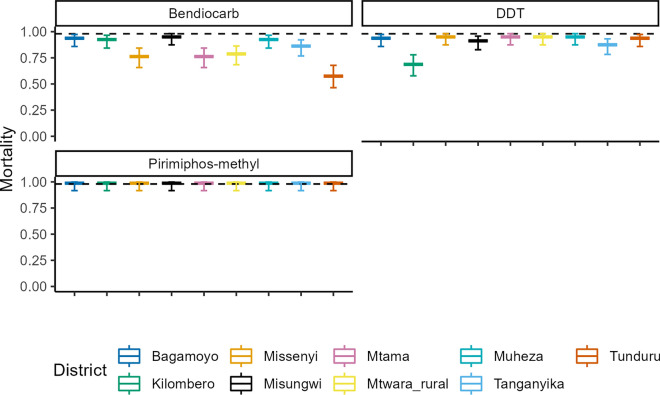
Insecticide resistance of *An. funestus* to bendiocarb, DDT and pirimiphos-methyl. The colours represent the different districts where the bioassays were conducted, the error bars represent the 95% confidence intervals of the predictions and the dotted line on the *y*-axis indicates a 98% mortality threshold

The 1-h knock-down (KD_1h_) to bendiocarb was greater than 80% in all locations, but DDT KD_1h_ less in Morogoro (55%) with complete knock-down in Mtama, Lindi region. Less KD_1h_ to pirimiphos-methyl was in Missenyi district (74%) but greater than 90% in all other districts. Phenotypic resistance to carbamates (bendiocarb) was observed in five of the nine study sites across the country, namely the southern sites of Mtama, Mtwara Rural and Tunduru, as well as the western site of Tanganyika and the north-western site of Misenyi, all showing less than 90% mortality. Conversely, high mortality rates, indicative of susceptibility to bendiocarb were observed in the eastern sites of Bagamoyo, Kilombero and Muheza, as well as in the northern site of Misungwi (Fig. 3). Resistance to the organochlorine, DDT was observed exclusively in the Kilombero district, in south-eastern Tanzania (mortality: 68% (57.7–77.9). This study also showed the universal susceptibility of *An. funestus* populations in all the study sites to the organophosphate, pirimiphos-methyl, as evidenced by the 24-h mortalities exceeding 98% (Fig. 3).

### Genetic markers of insecticide resistance in *An. funestus* in Tanzania

**Fig. 4:**
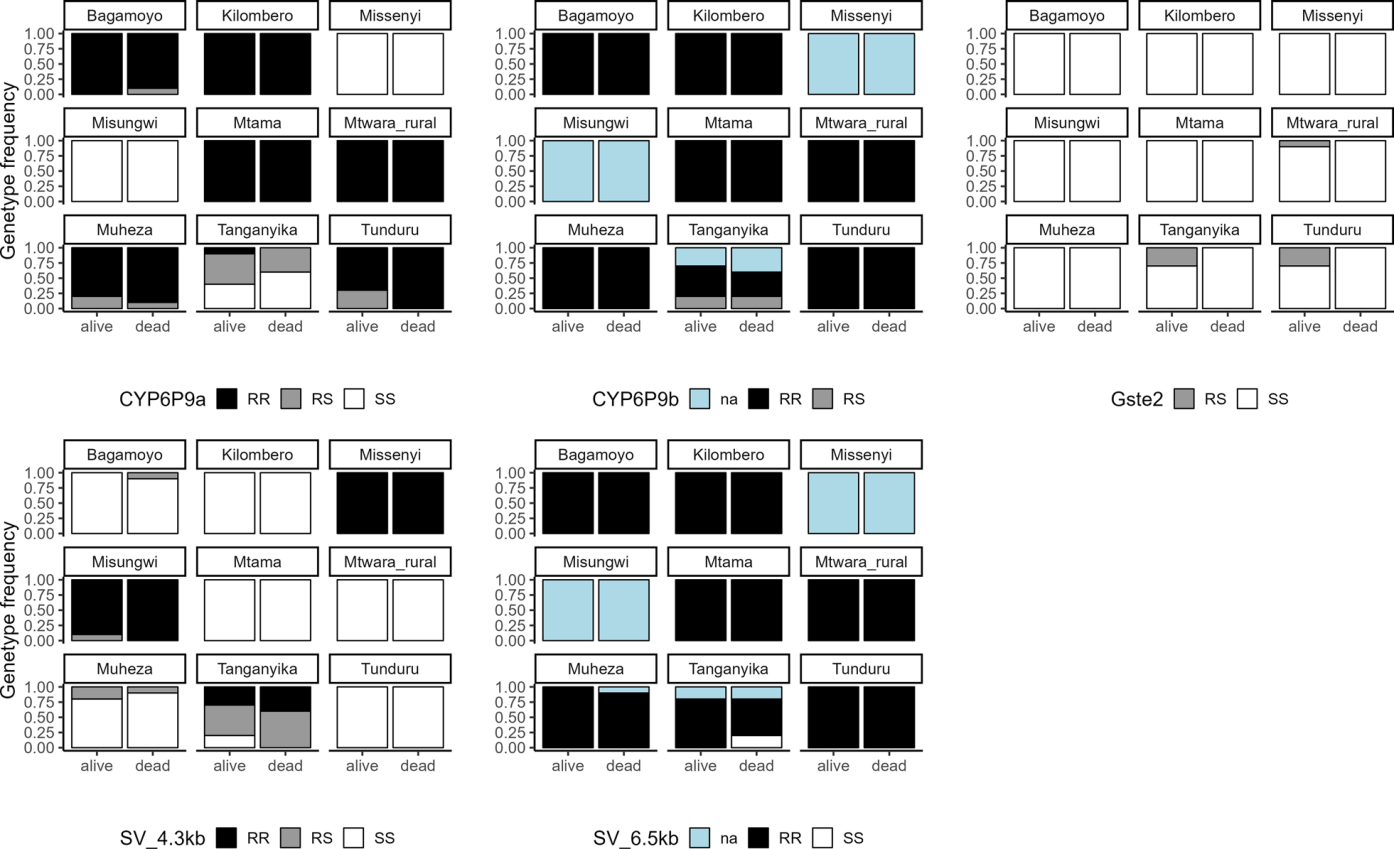
The distribution of metabolic resistance genotypes, *CYP6P9a*, *CYP6P9b* and L119F-*GSTe2,* SV 4.3 kb and SV 6.5 kb in phenotypically resistant (alive), and susceptible (dead) *An. funestus* across nine districts in Tanzania

The resistant allele *CYP6P9a*-R was prevalent in the eastern part of the country and was fixed (100% prevalence) in both phenotypically resistant (alive), and susceptible (dead) mosquitoes collected from Mtama, Mtwara rural and Kilombero districts, but was also observed in Tunduru, Bagamoyo and Muheza at very high frequencies (Fig. 4). In addition to the *CYP6P9a*-RR genotype, mosquitoes in Tunduru, Bagamoyo and Muheza districts also carried the heterozygous resistant genotype *CYP6P9a*-RS. This heterozygous form was also prevalent in the western district of Tanganyika. The *CYP6P9a*-RR genotype was completely absent in the north-western sites and was observed only at very low levels in Tanganyika in western Tanzania. Instead, *An. funestus* from Missenyi and Misungwi districts were all homozygous susceptible *CYP6P9a*-SS, with Tanganyika having all three genotypes; RR, RS and SS (Fig. 4). Similarly, *CYP6P9b*-R alleles were fixed in all districts except the north-western districts of Misungwi and Missenyi, where the gene did not amplify. In Tanganyika, both *CYP6P9b-*RR and *CYP6P9b*-RS genotypes were identified alongside a small number of non-amplifications (na) (Fig. 4). Contrastingly, the *L119F-GSTe2-*RR resistance genotype was absent in all study sites. Instead, *An. funestus* in six districts were homozygous susceptible for *L119F-GSTe2-*SS, while those in Tunduru, Mtwara rural and Tanganyika districts had the heterozygous haplotype of the gene *L119F-GSTe2-*RS in some surviving mosquitoes (Fig. 4).

In tests for the structural variants, it was observed that the homozygous resistant genotypes of SV 4.3 kb (RR) were near fixation and restricted to the north-western districts of Missenyi and Misungwi and were also found at low frequencies in the western district of Tanganyika (Fig. 4). Conversely, the homozygous resistant genotypes of SV 6.5 kb (RR) were widespread and fixed across most districts except the north-western districts of Missenyi and Misungwi, where the SV did not amplify in the PCR (i.e. na) (Fig. 4).

Further analysis revealed statistically significant geographic variations in the frequencies of *CYP6P9a* (*χ*^2^ = 172.62, *P* < 0.0005), *CYP6P9b* (*χ*^2^ = 16.04, *P* = 0.0135) and SV 4.3 kb (*χ*^2^ = 169.35, *P* < 0.0005). In contrast, no significant differences were observed for *L119F-GSTe2* (*χ*^2^ = 0.2324, *P* = 0.9116) or SV 6.5 kb (*χ*^2^ = 9.39, *P* = 0.1518) by district.

### Associations between genotypic and phenotypic expression of insecticide resistance

**Fig. 5:**
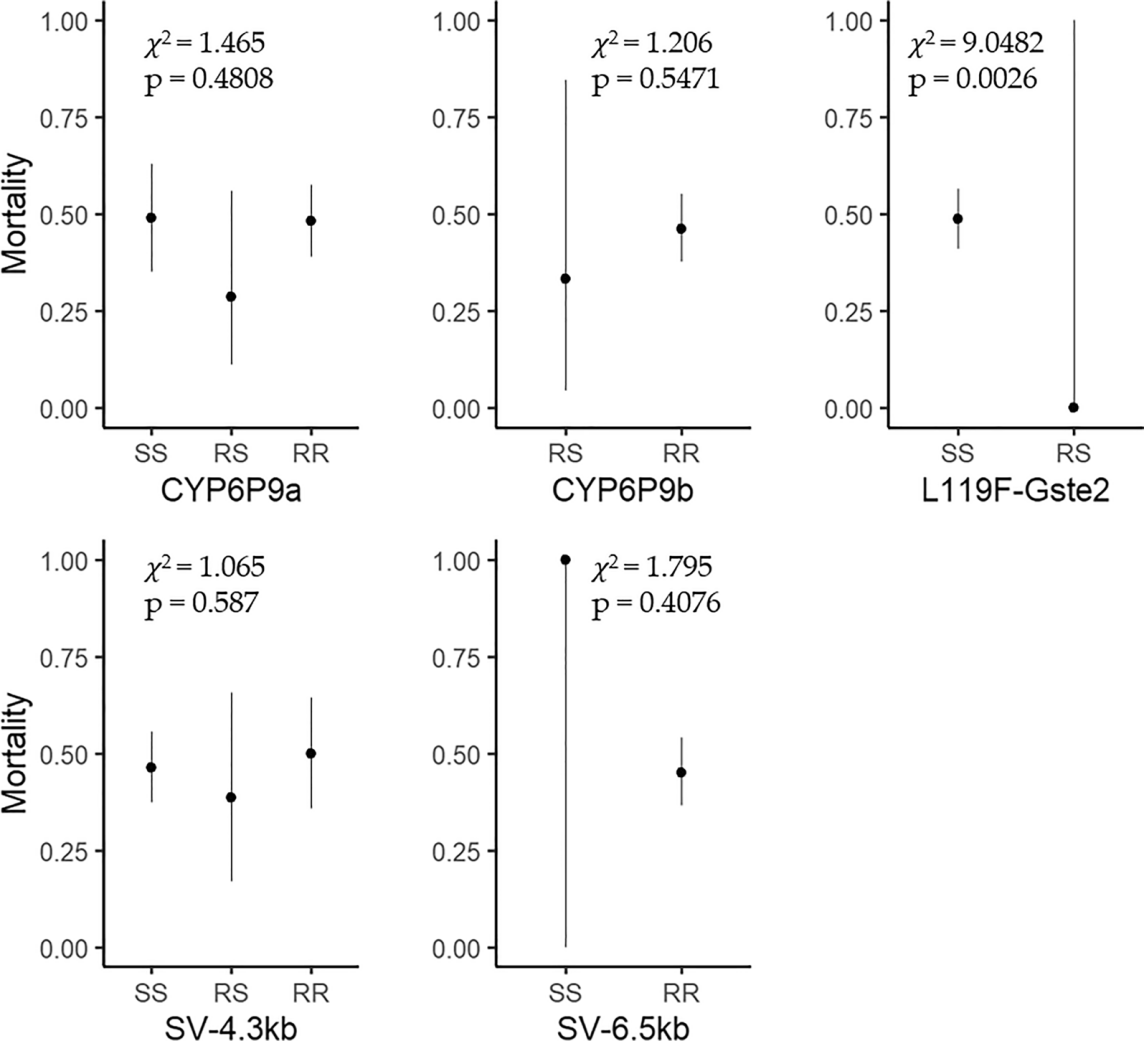
The association between metabolic resistance genes *CYP6P9a, CYP6P9b, L119F-GSTe2* and structural variants *SV 6.5 kb* and *SV 4.3 kb* with deltamethrin resistance phenotype; the *y*-axis indicates the predicted mortalities from each single genotype, with the point indicating the mean and line 95% confidence interval and the genotypes on the *x*-axis indicate SS, homozygous susceptible; RS, heterozygous resistant; and RR, homozygous resistant

Using equal samples of live and dead *An. funestus* following exposure to the candidate insecticides, and the subsequent genotyping as described above, we used generalised linear mixed models to test for associations between phenotypic expression of resistance (i.e. percentage surviving exposure) and the presence of specific genotypes (i.e. *CYP6P9a*, *CYP6P9b*, *L119F*-*GSTe2,* SV 4.3 kb and SV 6.5 kb). This analysis was done only for deltamethrin. The final model had only the *L119F-GSTe2* gene; indicating only variants of this gene were predictive of surviving lethal deltamethrin insecticide doses (*χ*^2^ = 9.0482, OR 2.5875, *P* = 0.0026) (Fig. 5). However, there was no significant association between either *CYP6P9a* (*χ*^2^ = 1.465, *P* = 0.4808), *CYP6P9b* (*χ*^2^ = 1.206, *P* = 0.5471), SV 4.3 kb (*χ*^2^ = 1.065, *P* = 0.587) or SV 6.5 kb (*χ*^2^ = 1.795, *P* = 0.4076) with survival to lethal deltamethrin doses (Fig. 5).

### Interactive map for visualisation of insecticide resistance

Since this was the first large-scale survey of insecticide resistance in Tanzania’s *An. funestus*, the findings could provide a critical foundation for future updates, preferably with a more granular coverage both geographically and temporally. More importantly, it could contribute to data-driven decision-making for resistance management when deploying vector control interventions. Therefore, to enhance practical application of the findings, we developed an interactive tool using R Shiny [36], enabling users to visualize the predicted insecticide resistance in *An. funestus* across Tanzania, and which could, in the future, be extended to other vector species. This user-friendly tool allows for the selection of various tested insecticides, displaying their predicted mortality rates on an interactive map. It highlights regional resistance patterns, aiding in the interpretation and application of our research data. The tool is accessible online at https://boydorr.gla.ac.uk/lucanelli/odero_ins_res_map/, and will be a valuable resource for researchers and public health practitioners working on malaria control.

## Discussion

*Anopheles funestus* is a major malaria vector in Tanzania and is particularly dominant in the north-western region and south-eastern regions [7, 8, 40]. Yet, its resistance to key public health insecticides has been patchy at best in comparison with the other malaria vectors such as *An. arabiensis* and *An. gambiae*, for which the resistance data are more commonly collected. This study provides the first large-scale contemporaneous dataset on both the genotypic and phenotypic expression of resistance in *An. funestus* to a range of insecticides, including two pyrethroids, deltamethrin and permethrin; an organochlorine, DDT; a carbamate, bendiocarb; and an organophosphate, pirimiphos-methyl.

The findings show widespread multiple resistance in wild *An. funestus* mosquito populations in mainland Tanzania and provides a basis for decision-making for national malaria control programs on the management of insecticide resistance and selection of appropriate interventions. The findings show pyrethroid resistance to be particularly common in *An. funestus* with high resistance intensities to deltamethrin in most locations. Similar pyrethroid resistance profiles in this vector have been shown in earlier but separate studies in the south and north of Tanzania [7, 8]. Continentally, phenotypic expression of pyrethroid resistance in *An. funestus* has been reported in other countries including Uganda [41], Mozambique [42], Malawi [43, 44], Ghana [23, 45] and Cameroon [46], with most countries still lacking bioassay data on this vector species. The synergistic effect of PBO, leading to the restoration of susceptibility in pyrethroid-resistant *An. funestus* mosquitoes were evident in all tested populations, pointing to the predominant involvement of metabolic-mediated resistance mechanisms. Malaria vectors of the *An. gambiae* complex has also shown similar restoration of pyrethroid-susceptibility with PBO [9]. In epidemiological studies evaluating ITNs treated with both PBO and pyrethroids in Tanzania [47] and Uganda [48], increased community protection against malaria was observed, compared to protection accrued with pyrethroid-only nets. Our results therefore add to the body of evidence on the efficacy of PBO-ITNs against pyrethroid-resistant malaria vector populations [49, 50].

The study also shows *An. funestus* across Tanzania to be susceptible to the IRS insecticide pirimiphos-methyl, unlike in *An. gambiae*, where resistance has been confirmed in parts of the country [51]. However, the observed resistance to the carbamate (bendiocarb) in parts of the country is concerning and should be factored in by malaria control programs if IRS campaigns are planned in the future. Though Tanzania has recently discontinued the use of IRS [52], this remains an important tool and could be especially useful in high transmission settings or emergencies to curb outbreaks. Phenotypic resistance to DDT was isolated to the Kilombero valley in south-eastern Tanzania and has been linked to the recent discovery of knockdown resistance (*kdr*) in *An. funestus* in the same location [53]. The *kdr* evolution in Kilombero is thought to be due to agricultural DDT contamination and a legacy of past extensive DDT stockpiles [53]. Without proactive management of resistance, the observed resistance situation in the country is likely to worsen, as similar insecticide classes are heavily used for agricultural pest control [54].

As vector control evolves in sub-Saharan Africa and as new or improved insecticidal interventions are introduced, it is crucial to monitor the genetic basis of insecticide resistance and track its evolution within and beyond Tanzania. The genotype frequencies of *CYP6P9a/b* and SV 6.5 kb IR genes have previously been estimated as low–moderate in Tanzania but fixed in other southern African countries [18, 19, 29]. Here, we found the resistant alleles of these three genes to be either fixed or near fixation across most localities in the eastern region of Tanzania, indicating how these mutants have recently selectively swept through *An. funestus* populations in Tanzania. The high frequencies of homozygous susceptible genotypes of *CYP6P9a-*SS in the northern Tanzania districts and the evidence of mixed genotypes in west of Tanzania (near Lake Tanganyika) could suggest a gene flow barrier in the country (possibly including landscape features such as the Great Rift Valley); or an ongoing south–north directional selection of the alternative homozygous resistant genotype *CYP6P9a-*RR arising from the southern Africa region [55]. This potential gene flow restriction could further explain the contrasting distribution of structural variants, with SV 4.3 kb fixed and restricted in the northern Tanzania districts (Missenyi and Misungwi) and SV 6.5 kb in the rest of the country. The cause of high non-amplifications of SV 6.5 kb and *CYP6P9b* in northern Tanzania is unclarified and warrants investigation. Nonetheless, it has been postulated to be due to the SV 4.3 kb [28]. Both structural variants have been demonstrated to exacerbate resistance in *An. funestus* by increasing the overexpression of *CYP6P9a* and *CYP6P9b* [28, 29]. Recent analysis had similarly linked such copy number variants with increased expression of the genes encoding metabolic resistance in *An. gambiae* and *An. coluzzii* [56, 57]. The lack of statistical association of *CYP6P9a/b,* SV 4.3 kb and SV 6.5 kb genes with pyrethroid resistance in this study is likely due to their already strong selection and fixation in the *An. funestus* populations in Tanzania. This could also be a stronger effect of alternate metabolic genes not analysed here but previously shown to be associated with *An. funestus* pyrethroid resistance in East Africa such as *CYP6M4, CYP9K1, CYP6M1* and *CYP6Z1* [11, 21]. Additionally, this observation could also have resulted from matching the number of samples genotyped, which did not consider the natural frequencies of these genes in the populations, hence lacking sufficient statistical power to detect a true effect.

*L119F-Gste2*, which confers cross-resistance to DDT and pyrethroids in *An. funestus* have previously been identified in West and Central Africa [24, 25, 27] and Uganda [41] is the only previous detection in Eastern Africa. However, findings of this study now establish that the mutation has now spread to Eastern Africa, with low frequencies of the *L119F-Gste2* resistance alleles detected in the Tanzanian districts bordering the Democratic Republic of the Congo (Tanganyika), Mozambique (Mtwara rural) and Malawi (Tunduru). The strong association observed between this genotype and deltamethrin resistance phenotypes is concerning and warrants close monitoring of its spread and evolution. Though it is not possible to conclusively describe the evolutionary path of this resistance allele in the region, its strong association with pyrethroid resistance could allude to a recent selection.

Overall, this study has highlighted the importance of broadening the resistance monitoring efforts to include all principal malaria vectors, including not just the members of the *An. gambiae* complex, but also *An. funestus*. This is particularly important in areas where this vector species dominates malaria transmission. One complementary outcome of this study was the extension to develop a user-friendly online platform for stakeholders, particularly scientists and public health officials to access the data. This platform lays the groundwork for informed decision-making regarding both the management of insecticide resistance and the actual deployment of effective interventions. The study also has the advantage of providing contemporaneous data from multiple sites with both genetic and phenotypic observations. However, it will be essential to regularly update this information, preferably with greater granularity in terms of both the geographical coverage at regional and sub-regional levels, and in terms of the temporal frequency of data collection.

One limitation of this study was the utilization of adult wild female mosquitoes of unknown ages in the bioassays. It is standard practice to utilize age-synchronized, 2–3-day-old mosquitoes in resistance bioassays to avoid ageing-related weaknesses [58] and environmental stresses. However, despite efforts to characterize the breeding habitats of *An. funestus* [13], this species is notoriously difficult to locate in most ecological settings, making it challenging to apply the standard approach. This also explains why resistance data on *An. funestus* have been largely missing in previous Tanzania’s sentinel surveys [9]. In this study, given the difficulties of finding the larvae, and the logistical challenges due to the geographical expanse covered, a decision was made to use adult samples. Therefore, as insecticide susceptibility increases with age in mosquitoes [59] we cannot completely rule out the potential confounding effect of ageing in our mortality data, which could have underestimated the resistant levels of our samples. However, this issue was mitigated by several steps including (a) using only unfed non-gravid adults, (b) holding the mosquitoes for at least 24 h to eliminate any mortality associated with mosquito handling, (c) normalizing excess mortalities using a set of controls, where natural mortality due to senescence was considered and (d) screening for both the genotypic and phenotypic expression of resistance in the mosquitoes.

Another significant constraint of this study was the potentially low statistical power to discern the associations between specific gene variants and phenotypic resistance. Given the sample sizes used, there was a predisposition towards detecting only variants with large effect sizes, while more subtle or moderate effects likely remained unidentified. Lastly, the study design and sample sizes did not permit an exploration of the cumulative or epistatic effects exerted by multiple gene mutations within the same individual mosquito. It is conceivable that a combination of mutations across the five genes investigated, rather than the impact of each mutation in isolation, could more accurately account for the variations observed in the phenotypic resistance. This complexity underscores the need for future studies to consider larger sample sizes and more sophisticated statistical and genetic analyses that can capture these nuances.

One area of improvement is that future tests should incorporate some of the newly approved insecticides for vector control, including the pyrrole chlorfenapyr, which is used in the dual-active Interceptor G2, nets and neonicotinoids, such as clothianidin, which is also approved by WHO for IRS [60]. Such expansion will provide essential data for the national malaria control program to guide their selection of interventions in the context of the evolving resistance patterns.

## Conclusions

This comprehensive study presents a cross-country landscape of insecticide resistance in *An. funestus*, a principal malaria vector in Tanzania. It marks an important step towards the understanding both the distribution and evolution of insecticide resistance mechanisms in Tanzania and will contribute significantly to evidence-based decision-making for improved vector control in the country. The study revealed widespread resistance to pyrethroids, albeit with notable restoration upon pre-exposure to the synergist piperonyl butoxide (PBO), underscoring the potential of PBO-impregnated nets in combating pyrethroid resistant populations of *An. funestus*. Additionally, the presence of resistance to carbamates, and in some sites, and the emergent resistance to DDT in the south-eastern part of the country, highlight the complexity of resistance in the vector and the significant threat to the existing arsenal of vector control insecticides. Fortunately, *An. funestus* populations across all study sites appear to remain susceptible to the organophosphate pyrimiphos-methyl, suggesting that this chemical, which is already widely used in agriculture, may be one of the few remaining options suitable for vector control, should the country reinstate the use of indoor residual spraying. The detection of genetic markers, including *CYP6P9a/b*, associated structural variants, and the mutation *L119F-GSTe2* across different districts, provide an explanation for genetic foundation underlying the observed phenotypic resistance patterns. Additionally, these genetic markers reveal some underlying gene flow patterns in the *An. funestus* populations and are suggestive of a barrier (potentially including the Great Rift Valley) as well as an ongoing south–north directional sweep of some genotypes, notably the *CYP6P9a-*RR. The findings of this study have been made readily accessible through an interactive online interface to make it directly applicable for insecticide resistance management and to emphasize the need for continuous surveillance and updates on this dataset. Given the significance of these insights for the development and deployment of effective vector control tools, further research is required on all major vector species, with a focus on increased granularity in the spatial and temporal analysis, to enable adaptive resistance management and improved malaria control in Tanzania and the region.

## Data Availability

No datasets were generated or analysed during the current study.

## Abbreviations

WHO: World Health Organization
IRS: Indoor residual spraying
ITNs: Insecticide treated nets
PBO: Piperonyl-butoxide
PCR: Polymerase chain reaction
*GSTe2*: Glutathione s-transferase epsilon 2
CDC: Centres for disease control and prevention
SV: Structural variant
*CYP450*: Cytochrome P450 gene family
